# A Variety of Leptothrix Discovered by Dr. W. D. Miller

**Published:** 1883-10

**Authors:** 


ARTICLE V.

### A VARIETY OF LEPTOTHRIX DISCOVERED BY DR. W. D. MILLER, AND NAMED BY HIM LEPTOTHRIX GIGANTEA

Dr. Miller had the kindness to send us a copy of the *Berichte Der Deutchen Botanischen Gesellschaft* containing a monograph about this parasite ; we give an abridged translation.

" In summer last year, Dr. Moller of the veterinary school at Berlin brought to me a dog suffering from Riggs' disease (pyorrhœa alveolaris), to have its teeth examined. On the scum covering the teeth there was a luxuriant development of a *spaltpilz* of gigantic dimensions, which was recognized in the course of the investigations as a new variety, and named leptothrix gigantea. The next question was if this organism did not also occur in the teeth of the carnivorous or phytophagous mamals, and this induced me to investigate their teeth in this direction, and I found that this fungus existed also in the mouths of sheep, cattle, hogs and horses.

The fungus appears usually in the shape of turfts or patches whose threads, similar to those of crenothrix, spread in different directions from their point of attachment. The appearance in patches seems to be due to the fact that such little groups of threads are developed from little lumps of cocci. I have seen that in the plainest manner in the scum of teeth from the mouth of a cat. One sees a little lump of round and oval cocci from which threads of varied length radicate in all directions. The older ones are segmented into baccilli and cocci, so that there can be no doubt of a connection between these three forms. The threads of some patches vary considerable in thickness, in a similar manner as perhaps crenothrix or beggiatoa. Some are very thin, others quite thick, and there are all transitions between these two. In the finer threads no difference of basis or top can be observed, but this is well marked in the larger ones.

The threads are sometimes straight, sometimes bent, and often quite regularly coiled; two or three are sometimes coiled together. In all those points leptothrix gigantea resembles beggiatoa alba and crenothrix Kuhniana. Segmentation may be observed sometimes in all the threads of some patches. As in leptothrix bucalis, the threads are very often segmented into bacilli and micrococci. All these could be seen without the use of re-agents in larger threads, mainly in the upper and middle parts. Very thin threads show the segmentation already very beautifully while alive, but to see them in the finest threads, staining substances have to be employed. Sometimes one can see all three forms in the same thread, transitions of bacilli to micrococci, but finally all is resolved into cocci. In the threads carrying partitions, the components, may they be bacilli or cocci, are discharged from them and collect in little heaps.

It is a special feature, chiefly of large threads, that the components show derivations from the older forms. The bacilli and cocci often swell up and become rounded so as to press against each other. Sometimes the bacilli swell to pear shape or, when touching the neighboring cell, develop bag-like excresences. In the older threads one can observe how the bacilli lying originally in one row, somehow become misplaced, so that they no longer coincide with the axis of the thread. If now the bacilli grow in the new direction, they grow side by side, and in consequence of this occurrence the thread, which was originally uniform, appears more or less strikingly broken. We have seen that the form of cocci may originate in the first part by transverse segmentation. If such segmentations occur in small threads, the cocci show of course very small diameters; but if they occur in large threads the diameter is much larger, too. From the large cocci by progressive divisions smaller cocci may be produced. This could best be observed in the form of leptothrix gigantea that occasionally occurs in the teeth of pigs. There occurs in the first place a transverse segmentation of a large cocous so that this is divided into two disks, but then every disk, is divided lengthwise, so that the originally large coccus is divided by divisions in two directions of the space into four smaller ones. Sometimes such a formation can be seen for the whole length of a thread. At the beginning cube-shaped, those cocci afterwards become loose and move so that the original shape becomes obliterated. These divisions in two directions can often be proved by the use of staining materials.

The spinal form occurs in the larger and smaller threads in the three modifications of spirillium, vibrio, and spirochæte, all transitions from spirillium, to vibrio, and from vibrio can sometimes be seen on the same thread. Very often the spiral forms show no trace of segmentation, and only by the use of staining materials can the division be made visible.

I presume that the fine forms of spirilla found on the scum covering the teeth in man, which has been designated until now as *spirochæte dentium*, might originate by segmentation of the long threads.

Spirochæte dentium has been considered until now to be a unicellular structure, and although I took the greatest possible pains, I could not discover any segmentation in bacilli and cocci, but by applying the method of W. Zopf, I have been able to prove beyond doubt the segmentation in parts of unequal size. These pieces agree in size and shape exactly with the " dental bacterium " as observed in the mouth. To prove this segmentation, the strongest lenses witn oil immersion and Abbe's illumination are needed. The specimen ought not to be colored very intensively.

A great number of very fine drawing accompany the excellent monograph.—*New. Eng. Jour. of Dentistry.*

